# Diagnostic utility of novel combined arrays for genome-wide simultaneous detection of aneuploidy and uniparental isodisomy in losses of pregnancy

**DOI:** 10.1186/1755-8166-7-43

**Published:** 2014-06-24

**Authors:** Stefanie Bug, Beate Solfrank, Felizitas Schmitz, Jana Pricelius, Mona Stecher, Andrew Craig, Marc Botcherby, Claudia Nevinny-Stickel-Hinzpeter

**Affiliations:** 1 synlab Medizinisches Versorgungszentrum Humane Genetik München, Lindwurmstraße 23, D-80337 Munich, Germany; 2 BlueGnome Ltd, An Illumina Company, Cambridge, UK

**Keywords:** Product of conception, Early pregnancy loss, Aneuploidy, Loss of heterozygosity, Combined microarray, Genetic diagnostics

## Abstract

**Background:**

This proof-of-principle study demonstrates the usefulness and robustness of a novel array based method for the elucidation of genetic causes underlying early pregnancy loss. A combined microarray utilizing comparative genomic hybridization and single nucleotide polymorphism detection (CGH + SNP) was used for parallel genome-wide identification of copy number and heterozygosity status of 70 products of conception. Results of samples with previously determined aneuploidies were juxtaposed to those of a second cohort appearing normal after routine genetic diagnostics.

**Results:**

All chromosomal imbalances were confirmed, in one sample of the aneuploid panel additional monosomy X was discovered. Genome-wide uniparental disomy causing a complete hydatidiform mole was identified in another sample. No specimen featured microaberrations of obvious clinical relevance. Among cases with presumable euploidy, one microdeletion and a single region of homozygosity were assigned unclear clinical significance.

**Conclusions:**

The results prove the utility of combined imbalance and homozygosity mapping for routine workup of these challenging specimens. Moreover parallel screening at submicroscopic resolution facilitates the detection of novel genetic alterations underlying spontaneous abortion.

## Background

About 8 to 20% of clinically confirmed pregnancies abort spontaneously in the first trimester [1,2]. About half of these early abortions can be explained by chromosome aneuploidy. Non-genetic contributing factors include immunological disorders, thyroid or blood coagulation conditions, acute or chronic diseases of the mother, stress or environmental influence. In order to determine the best medical treatment for all possible causes underlying embryonic demise, genetic analyses of products of conception (POCs) were introduced to diagnostic routines decades ago. Since then, clinical geneticists have been facing two major diagnostic concerns: First of all, mosaic constellations in embryo or placenta remain a profound analytical problem related to POC specimen [3,4]. Mosaics can only be reliably assessed by comparative analysis of different embryonic and/or chorionic cell types, which is usually not feasible in a diagnostic setting [5]. Moreover, there always is the suspicion of mutations that lie beneath detection limits and resolution of routinely applied protocols. Studies conducted in this respect aimed at the designation of clinically relevant segmental imbalances (deletions or duplications) and uniparental disomy (UPD) [6-10]. Molecular karyotyping by array based comparative genomic hybridization (aCGH) or single nucleotide polymorphism (SNP) detection enabled genome-wide profiling at even higher resolution [11-19]. The results showed in unison that segmental chromosomal aberrations seem non-recurrent and sporadic events in spontaneous abortions.

Most often distal gains or losses arise from the presence of chromosome derivatives from a balanced translocation carrier parent. In this clinical context, the introduction of precise imbalance mapping meant significant progress. In other cases the pathogenic relevance of microimbalances was sometimes hard to evaluate at the state of knowledge on the human genome. The same applied to genomic segments displaying UPD, which in general can be associated with genomic imprinting disorders and autosomal recessive conditions [20]. UPD often shows in mosaic constellations [21]. Potentially, all these features could contribute to early fetal loss. The occurrence of UPD increases with elevating aneuploidy risks due to advancing maternal age [22]. Systematic studies concordantly reported low frequencies of UPD in POCs, but discussed UPD as a possibly underestimated pathogenic factor for spontaneous abortion due to detection limits of available methods [23-27]. UPD comprises the concepts of uniparental heterodisomy (UPHD) and isodisomy (UPID). However, the occurrence of either in POCs is unknown [28].

SNP profiling enables the identification of UPID via homozygosity mapping. The analytical challenge lies in the differentiation of pathogenic regions displaying absence of heterozygosity (AOH) from benign long contiguous stretches of homozygosity occuring frequently in the human genome [29,30].

The major advantage of array based cytogenomic approaches is that they overcome cell culture failure and- most often- overgrowth by maternal cells [31]. Thus, microarray screening has been repeatedly suggested as first tier test in the analytical workup of missed abortions [32-34].

**Figure 1:**
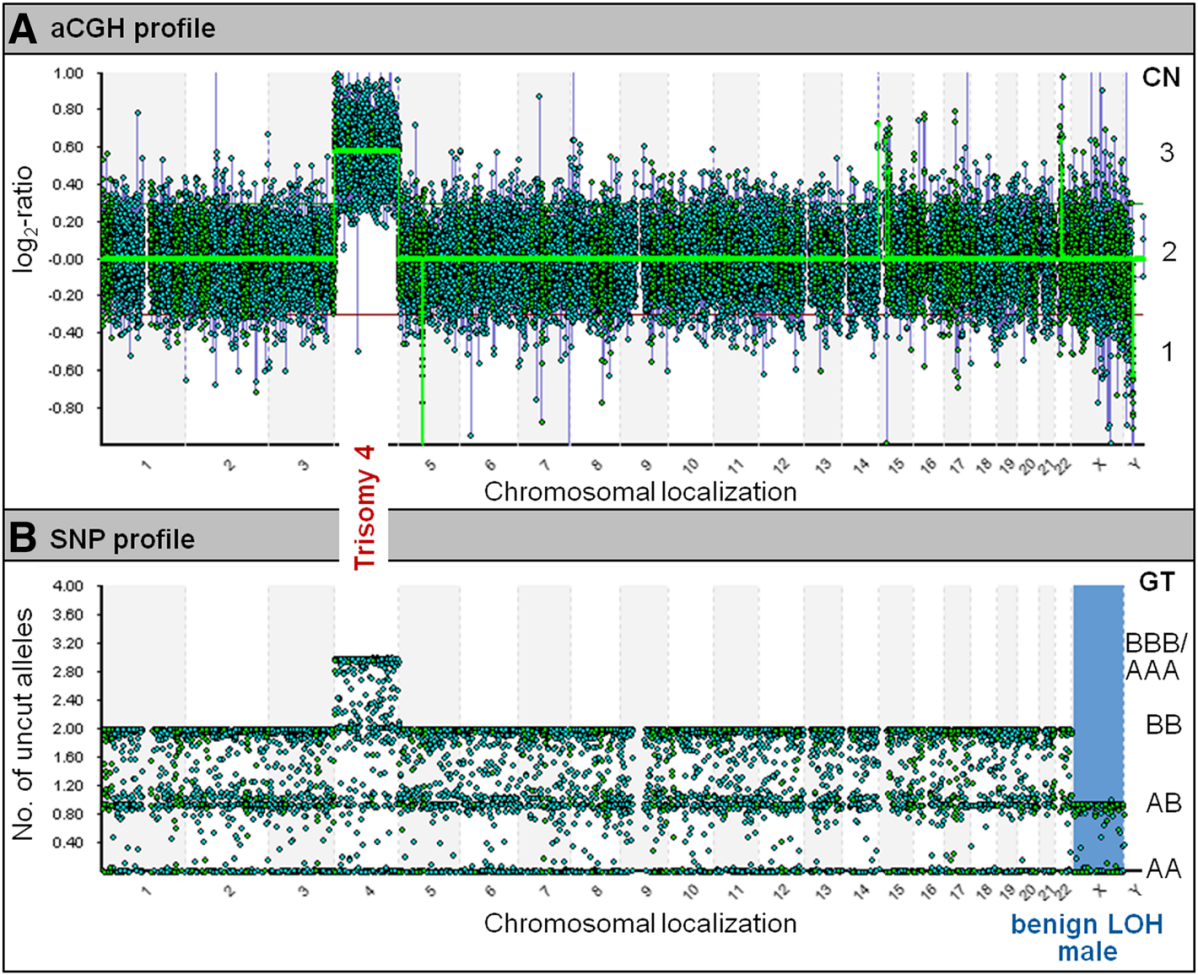
**Genome-wide CGH and SNP hybridization profiles for a sample displaying trisomy 4.** Panel **A**: aCGH data, CN: copy number, Panel **B**: SNP data, GT: genotype.

Against this background, we customized a novel CGH + SNP-8x60K microarray for POC analyses. A comparison of this approach to conventional platforms is given in Table 1. Representative hybridization profiles of the novel platform are displayed in Figure 1. The combined detection featured by CGH + SNP-arrays promised a superior robustness with regard to samples of varying quality and thus a possible advantage for POC analyses. With regard to this, the presented feasibility study addressed two key questions: Firstly, if a combined array approach at this resolution was optimal for the reliable detection of known genomic aberrations underlying first trimester fetal demise. Secondly, whether this platform unmasked recurrent small copy number associated or copy number neutral aberrations that could be causative of pregnancy loss in specimens of normal karyotype. To evaluate its utility in a diagnostic setting, the novel test was applied to a representative probe panel from routine diagnostics in our facility.

**Table 1:** Juxtaposition of conventional CGH- and SNP-array techniques to the combined approach chosen in this study

	**Conventional aCGH**	**Conventional SNP-array**	**Novel CGH + SNP-array**
**Array design**	One target consists of one 60mer oligonucleotide	Allele-specific 60mer olionucleotide targets, arranged in quartets differing in a single base each are arranged for SNP-detection	CGH + SNP-arrays contain both kinds of probes
**Type of aberrations targeted**	Copy number aberrations	Copy number and copy number neutral deviations (LOH)	Copy number and copy number neutral deviations (LOH)
**Mode of hybridization**	Comparative hybridization with a reference sample	Sample hybridization only	Comparative hybridization with a reference sample
**Mode of detection/ algorithm settings**	Copy number determination by comparison with a diploid reference sample	Determination of heterozygosity status by measuring of A- and B-alleles and *in silico* comparison to reference sample sets, deduction of copy number status from that	Combined detection: Only one allele is measured, the total number of alleles at given loci are estimated from CGH-probe signals

## Results

High molecular weight genomic DNA was successfully extracted from all 70 POC-samples. In 12 of them protein contamination could not be completely removed by ethanol precipitation resulting in A260/280 < 1.8 (1.740-1.795). Hybridization of respective DNA resulted in notably noisier imbalance profiles (derivate log ratio spread value (DLRS) = 0.16-0.29). However a lower DNA quality was not associated with a general dropout of SNP data (informative SNPs = 32.5-90.6%). Samples with A260/280 > 1.8 and A260/230 > 2 displayed a DLRS <0.2 and more than 80% informative SNPs. Although it was possible to reliably evaluate profiles of lower quality as well, these parameters were determined as optimal for analyses on the novel array platform. The design comprised a single region in 9q22.32, in which coincidental combination of oligonucleotide CGH probes with suboptimal hybridization properties lead to frequent false positive indication of a 47 kb genomic loss.

Microarray data are available in the ArrayExpress database (http://www.ebi.ac.uk/arrayexpress) under accession number E-MTAB-1725. An overview is given in Table 2. Array experiments confirmed all previously known aneuploidies. Additionally, monosomy X was detected in a specimen with 47,XX,+12 karyotype after routine cytogenetics. It had displayed sparse proliferation in culture. Although a low log_2_-ratio of chromosome X in the respective profile did not suggest it, array and karyotyping findings did not exclude the possible presence of a placental mosaic. For two near-triploid specimen with XXY-constellation, triploidy was indicated by low log_2_-ratios of gonosomes in aCGH-profiles. Remarkably, in one of them additional loss of chromosome 11 was missed, whereas an additional gain of chromosome 21 in the other sample was called correctly. It was speculated whether different aberration sizes or types of imbalances mislead the algorithm to bivalent copy number determination. Tetraploidy in the two samples with 92,XXXX karyotype was missed.

**Table 2:** Comparison of results obtained in the current study and results from diagnostic routine

			**POC**	**Result after routine diagnostics (obtained by karyotyping, FISH or aCGH)**^**b**^	**Result obtained in the current study by aCGH + SNP**^**b**^	**Congruency**
**Samples with known aneuploidy**	Native villi	Proliferation in cell culture	1	45,X	arr (X)×1	Yes
			2	45,X	arr (X)×1	Yes
			3	45,X	arr (X)×1	Yes
			4	45,X	arr (X)×1	Yes
			5	47,XX,+7	arr (7)×3	Yes
			6	47,XX,+7	arr (7)×3	Yes
			7	47,XX,+10	arr (10)×3	Yes
			8	47,XX,+12	arr (10)×3	Yes
			9	47,XX,+12	arr (12)×3,(X)×1	Yes, additionally monosomy X detected
			10	47,XX,+14	arr (14)×3	Yes
			11	47,XX,+15	arr (15)×3	Yes
			12	47,XX,+15	arr (15)×3	Yes
			13	47,XX,+16	arr (16)×3	Yes
			14	47,XY,+16	arr (16)×3	Yes
			15	47,XY,+16	arr (16)×3	Yes
			16	47,XX,+18	arr (18)×3	Yes
			17	47,XY,+20	arr (20)×3	Yes
			18	47,XY,+21	arr (21)×3	Yes
			19	47,XX,+21	arr (21)×3	Yes
			20	47,XX,+22	arr (22)×3	Yes
			21	47,XX,+22	arr (22)×3	Yes
			22	47,XY,+22	arr (22)×3	Yes
			23	48,XX,+6,+10	arr (6)×3,(10)×3	Yes
			24	48,XX,+7,+16	arr (7)×3,(16)×3	Yes
			25	48,XY,+14,+20	arr (14)×3,(20)×3	Yes
			26	48,XX,+18,+22	arr (18)×3,(22)×3	Yes
			27	49,XX,+13,+16,+21	arr (13)×3,(16)×3,(21)×3	Yes
			28	68,XXY,-11	arr (X)×1 ~ 2	No, gonosome profile indicates polyploidy, but loss of chr. 11 was missed
			29	70,XXY,+21	arr (21)×3,(X)×1 ~ 2	Yes
			30	92,XXXX	arr (1–22,X)×2	No, tetraploidy was missed
			31	92,XXXX	arr (1–22,X)×2	No, tetraploidy was missed
		Cell culture failure	32	arr (4)×3^c^	arr (4)×3	Yes
			33	arr (15)×3^c,d^	arr (15)×3	Yes
			34	arr (16)×3^c^	arr (16)×3	Yes
			35	arr (22)×3^c^	arr (22)×3	Yes
**Samples with presumptive euploidy**	Native villi	Proliferation in cell culture	36	45,XY,rob (13;14) (q10;q10)	arr (1–22)×2,(XY)×1	Yes
			37	46,XX	arr (1–22,X)×2 hmz	Yes, additionally hydatidiform mole detected
			38	46,XY	arr 2p24.3 (12,849,885-15,823,789)×1	Yes, additionally CNC detected
			39	46,XX	arr 6q23.2q24.31 (131,723,146-147,738,560)×2 hmz	Yes, AOH of uncertain clinical significance detected
			40	46,XY	arr 6q23.3q25.1 (135,836,902-150,015,258)×2 hmz	Yes, but false positive AOH call
			41	46,XY	arr (1–22)×2,(XY)×1	Yes
			42	46,XY	arr (1–22)×2,(XY)×1	Yes
			43	46,XY	arr (1–22)×2,(XY)×1	Yes
			44	46,XY	arr (1–22)×2,(XY)×1	Yes
			45	46,XY	arr (1–22)×2,(XY)×1	Yes
			46	46,XY	arr (1–22)×2,(XY)×1	Yes
			47	46,XY	arr (1–22)×2,(XY)×1	Yes
			48	46,XY	arr (1–22)×2,(XY)×1	Yes
			49	46,XY	arr (1–22)×2,(XY)×1	Yes
			50	46,XY	arr (1–22)×2,(XY)×1	Yes
			51	46,XX	arr (1–22,X)×2	Yes
			52	46,XX	arr (1–22,X)×2	Yes
			53	46,XX	arr (1–22,X)×2	Yes
			54	46,XX	arr (1–22,X)×2	Yes
			55	46,XX	arr (1–22,X)×2	Yes
			56	46,XX	arr (1–22,X)×2	Yes
			57	46,XX	arr (1–22,X)×2	Yes
	Cultured villi		58	46,XX	arr (1–22,X)×2	Yes
			59	46,XX	arr (1–22,X)×2	Yes
			60	46,XX	arr (1–22,X)×2	Yes
			61	46,XX	arr (1–22,X)×2	Yes
			62	46,XX	arr (1–22,X)×2	Yes
			63	46,XX	arr (1–22,X)×2	Yes
			64	46,XX	arr (1–22,X)×2	Yes
	Native villi	Cell culture failure	65	nuc ish (DXZ1x2,D18Z1x2), (RB1x2,D16Z1x2,D18Z1x2,DSCRx2,BCRx2)	arr (1–22,X)×2	Yes
			66	nuc ish (DXZ1x1,DYZ3x1,D18Z1x2), (RB1x2,D16Z3x2,DSCRx2,BCRx2)	arr (1–22)×2,(XY)×1	Yes
			67	arr (1–22,X)×2^e^	arr (1–22,X)×2	Yes
			68	arr (1–22)×2,(XY)×1^c^	arr (1–22)×2,(XY)×1	Yes
			69	arr (1–22)×2,(XY)×1^c^	arr (1–22)×2,(XY)×1	Yes
	NF^a^		70	arr (1–22)×2,(XY)×1^f^	arr (1–22)×2,(XY)×1	Yes

^a^NF: native fetal.

^b^according to ISCN 2013 [35].

^c^4x180K (Agilent Technologies, Santa Clara, USA).

^d^8x60K (Agilent Technologies, Santa Clara, USA).

^e^24sure (BlueGnome, Ltd., Cambridge, UK).

^f^4x44K (Agilent Technologies, Santa Clara, USA).

In the panel of POCs with presumptive normal karyotype one aberration of obvious clinical significance was unmasked: AOH affecting all chromosomes indicated genome-wide UPID and thus the formation of a complete hydatidiform mole in this patient. Interestingly, the algorithm called only 2 heterozygous SNPs as informative (0.03%), which were in fact false calls for a diploid genome build of two identical haploid paternal chromosome sets.

The novel combined microarray did not discover any alterations clearly associated with common microdeletion, −duplication or UPD-related syndromes. Separate from whole chromosome aneuploidy, numbers of detected microimbalances in aneuploid and euploid panels were comparable and varied from 3 to 13 deviations per sample (0.2 kb-4 Mb). Imbalances occurring frequently in both groups overlapped with known polymorphic chromosomal regions. Hybridizations of high quality genomic DNA correlated with low numbers of detected irregularities.

In one sample with normal karyotype a heterozygous loss of 3 Mb was revealed targeting the chromosomal region of 2p24.3. It comprised four genes (*TRIB2*, *FAM84A*, *NBAS* (*NAG*) and *DDX1*), whose molecular functions did not imply an obvious cellular cause for miscarriage. However, potential pathogenicity was accredited to the fact that the proximal breakpoint of the deletion mapped close to *MYCN*. Its gene product, the N-Myc protein, is a key regulator of skeleton morphogenesis and organogenesis during embryogenesis [36,37]. Breakpoint mapping by FISH with locus specific probes failed due to insufficient cell integrity of the frozen specimen. Thus, it could not be verified, whether the deletion affected regulatory elements of *MYCN*. Parental samples were unavailable, so the loss was classified as CNC (potentially pathogenic).

AOH counts per genome varied from 1 to 32. Intervals ranged from 8.4 to 24.2 Mb in size. No POC exhibited an excessive degree of AOH indicating a high grade of parental consanguinity [38]. Segments with AOH occurring in both panels were not assigned any clinical relevance and evaluated as benign.

In two euploid samples AOH mapped to 6q24, overlapping with the UPD critical segment for transient neonatal diabetes mellitus and at the same time with a reported region of biparental apparently benign homozygosity [39]. One of them spanned 16 Mb in 6q23.2q24.3, the other one 14.2 Mb in 6q23.3q25.1. Follow-up by STR was possible in the latter case. It revealed heterozygosity and thus, a technical artifact.

## Discussion

The comprehensive analysis of POCs always represents a challenge. It is usually met by a workflow of cytogenetic, molecular cytogenetic and molecular genetic methods depending on their availability in the diagnostic lab. This study demonstrates that the established microarray platform is suitable for the reliable simultaneous detection of imbalances and AOH in abortion material. It facilitates the unification of diagnostic steps in various laboratory workflows, which helps to significantly reduce costs.

### Technical utility

A major advantage of molecular karyotyping is its independency from proliferation of starting material in cell culture. It also overcomes *in vitro* selection against certain aneuploidies, cultural artifacts or maternal cell overgrowth. Maternal contamination of the tissue used for DNA extraction cannot always be precluded even after meticulous preparation of chorionic villi. There are further material-related aspects to consider in diagnostics, especially with regard to mosaicism: While DNA is extracted from all cell types present in the villi sample, karyotyping assesses mainly well-proliferating cells from the extraembryonic mesoderm, and FISH cells of the cytotrophoblast [5].

Good quality of genomic DNA reflected in excellent DLRS-values for aCGH, but did not guarantee a high percentage of informative SNP probes. We suspect postmortem DNA modification and/or degradation resulting in incomplete or faulty restriction digestion not detectable by AGE. Alternative digestion assays with isoschizomers lacking any methylation sensitivity did not alter SNP profiles notably (data not shown).

Our observations suggest that SNP calls were more readily affected by DNA quality than CGH calls. We therefore evaluate the use of a combined CGH + SNP approach superior to hybridizations to sole SNP arrays, because copy numbers are “determined” via more robust CGH, not by SNP based copy number “calling”.

SNP detection on the combined platform is substantially different from that on sole SNP arrays. Instead of direct measurement of both alleles at one locus, only one allele is assessed by aCGH + SNP. Restriction digestion is used to selectively remove one allele. Measurement of the remaining non-digested allele and the copy number state determined by aCGH are used to constitute the presence of heterozygosity. This means the combined chip does not have an equivalent statistic to the B-allele frequency, which is the standard way in which a triploid sample would be detected by a conventional SNP array. Since aCGH is not able to detect polyploidy, the linked SNP analysis proceeds assuming the sample is diploid. Thus, the identification of triploid samples in this study succeeded only for those, in which the presence of a Y chromosome lead to low log_2_-ratios of gonosomes. For the same reason, combined cytogenomics did not overcome the inability of aCGH to distinguish samples of 46,XX karyotype from tetraploid samples. Like conventional aCGH, the novel method cannot detect ratio changes of sample to reference extending genome-wide.

The combination of aCGH and SNP approaches does not fundamentally emend the detection of mosaicism and chimerism. In contrast to sole SNP arrays, the method is blind to mosaics of both UPHD and UPID [40]. With regard to mosaic constellations of imbalances, low log_2_-ratios indicating different genomes in the study panel were due to known maternal contamination. Based on our experience with medium resolution aCGH, most often cytogenetically proven placental or fetal mosaics display either as distinct gains or losses or not at all. In routine diagnostics we report them only after verification by karyotyping or FISH.

We point out that the specimen with the Robertsonian translocation was assigned to the panel of normal samples. Euploidy was confirmed by array analysis. Nevertheless we emphasize in diagnostic reports that mosaicism of low level trisomy cannot fully be excluded.

### Diagnostic utility

We established a robust assay for the rapid detection of aneuploidy and UPID in a one step analysis. Genome-wide UPID is associated with complete hydatidiform moles. Hydatidiform moles are at high risk of developing into choriocarcinomas and demand specific medical treatment.

Results of this pilot study show that the chosen probe resolution is reasonable for oligonucleotide detection of segmental aberrations in POCs, because it does not lead to excessive calling of microalterations, which then have to be evaluated with regard to their clinical significance. At medium resolution, partial chromosome imbalances due to derivative chromosomes from balanced reciprocal translocation carrier parents will reliably be picked up since those are usually detectable at the microscopic level. Apart from this, microduplications and microdeletions unambiguously associated with early pregnancy loss seem sporadic events. The clinical evaluation of CNCs will become easier with the ongoing understanding of functional elements in the human genome.

For the evaluation of partial AOH we consider it reasonable to apply the UPD thresholds determined in postnatal diagnostics as a guideline: due to steric reasons in recombination, UPD usually affects stretches of 20 Mb or wider in interstitial and appr. 10 Mb in telomeric regions [39]. Segments of homozygosity smaller than this are more likely to represent benign AOH. As knowledge of epigenetic effects such as imprinting increases, homozygosity mapping will become an important tool in the elucidation of pathogenetic mechanisms in abortions. There is an urgent need for valid databases on UPD effects on fetal demise. Now that it becomes technically possible to generate these data, our platform being a part thereof, they will hopefully arise. Strict differentiation between UPHD and UPID in scientific and medical reports will greatly promote this. Homozygosity profiling as it is presented here, will only detect UPID. For UPHD, both parents have to be included in the array screening as well. In diagnostics this is very expensive, but feasible (though not with current algorithm options of this platform). However, at the current state of medical science it is clear that segmental or complex UPD arises only from co-occurrence of meiotic or mitotic recombination, abnormal segregation, and subsequent correction [41]. Therefore the risk of recurrence is negligible versus the risk of invasive prenatal diagnosis in a future pregnancy.

On the long road towards genetic analysis at base pair resolution, we present a profound diagnostic method that at the same time allows further insight into pathogenic molecular mechanisms resulting in spontaneous abortions. The clinical benefits of POC-analyses remain unquestionable: they permit accurate genetic counseling and the estimation of recurrence risks. Moreover they help to determine the best medical treatment to fulfill the couples’ desire for a healthy child.

## Conclusion

We suggest genome-wide CGH + SNP profiling as a first tier test for simultaneous detection of copy number associated and copy number neutral aberrations underlying spontaneous abortion. Although polyploidy and structural rearrangements have to be assessed in further diagnostic steps, the peculiar advantages of this novel cytogenomic method are independency from cell culture proliferation, reliable detection of chromosomal aneuploidy and pathogenic microimbalances as well as genome-wide UPID underlying hydatidiform moles.

## Methods

This study was done on 70 samples of fetal tissue or chorionic villi from early pregnancy losses remaining after genetic diagnostics (native n =63, cultivated n =7). Written informed consent was obtained from all patients for the use of material from products of conception after completion of genetic diagnostics and further use in this study. Ethical approval was granted for this study by an interdisciplinary institutional reviewer board of medical and scientific experts of the participating centers. From conventional cytogenetics, karyotypes were known for 60 POCs. Due to cell culture failure, 10 samples were assessed by molecular cytogenetics: 2 of them by fluorescent in situ hybridization (FISH) with probes targeting chromosomes most commonly affected by aneuploidies (13, 16, 18, 21, 22, X, Y) and 8 samples by genome-wide aCGH. If nondistinctive, fetal origin of material had been verified by short tandem repeat marker analysis (STR). Thus, the panel comprised 35 samples previously determined as aneuploid (31 by karyotyping and 4 by aCGH) and 35 samples that appeared euploid after diagnostic routine testing (29 by karyotyping, 2 by FISH and 4 by aCGH, protocols available upon request).

Specimens of dissected fetal tissue or cultured cells were thawed and washed three times in phosphate buffered saline (Invitrogen, Carlsbad, USA). Genomic DNA was extracted utilizing the Maxwell®16 LEV Blood DNA kit (Promega Corp., Madison, USA), MagNA Pure kit (Roche, Grenzach-Wyhlen, Germany) or QIAamp DNA Blood Mini kit (QIAGEN, Hilden, Germany) according to respective manufacturers’ protocols. Concentration and quality of genomic DNA was assessed spectrophotometrically (NanoDrop® ND 1000, peqlab Biotechnologie GmbH, Erlangen, Germany). If necessary, ethanol/sodium acetate precipitation was performed. High molecular weight of genomic DNA as well as complete restriction digestion were verified by agarose gel electrophoresis (AGE) according to standard laboratory protocols.

Sample-DNA was hybridized to custom CGH + SNP-8×60K microarrays (BlueGnome Ltd., Cambridge, UK). The design was genome-wide, but targeted known disease genes and regions (design identification: MUNCH005). It was made up of 59 090 60mer oligonucleotides mapped to the human genome according to the Genome Reference Consortium Human Build 37 of February 2009 (GRCh37/hg19). The practical resolution for aCGH was appr. 200 kb corresponding to three consecutive deviating probes with average spacing of 66 kb. The design comprised 18% SNP-probes enabling the detection of stretches of homozygosity of 13 Mb or wider. Respective male or female genomic DNA of known haplotype derived from single individuals displaying no clinical phenotype was used as reference (SNPRef DNA, commercially not available, BlueGnome Ltd.). Wherever applicable, samples were cohybridized with sex-matched references according to the CytoChip Oligo reference manual v 1.8 (BlueGnome Ltd.). Slides were scanned in double pass mode at 3 μm resolution with a G2565CA microarray scanner (Agilent Technologies, Santa Clara, USA). Data were analyzed with software BlueFuseMulti versions 2.5 to 3.0 (BlueGnome Ltd.). In addition, aCGH and SNP-profiles were inspected manually and simultaneously in a 3 Mb sliding window. Sex-mismatch derived copy number deviations and benign AOH of chromosome Y in male samples served as internal controls. Detected imbalances were assigned either benign copy number variation (CNV), pathogenic copy number alteration (CNA) or copy number change of uncertain clinical significance (CNC) [42]. Homozygous segments were rated as benign, pathogenic, or AOH of uncertain clinical significance. The following online databases were used in the evaluation process: Database of Genomic Variants Beta (DGVbeta, http://dgvbeta.tcag.ca/dgv/app/home), Decipher (DECIPHER, http://decipher.sanger.ac.uk), International Standards for Cytogenomic Arrays (ISCA, http://www.iscaconsortium.org) and the UPD-database of the University Medical Centre of Jena, Germany (http://ssmc-tl.com/Start.html).

For verification of homozygosity in the 6q24 chromosomal region, STR analyses were carried out utilizing the PowerPlex® ES system (Promega Corp).

## Abbreviations

aCGH: Array based comparative genomic hybridization; AGE: Agarose gel electrophoresis; AOH: Absence of heterozygosity; CGH: Comparative genomic hybridization; CNA: Copy number aberration; CNC: Copy number change; CNV: Copy number variant; DLRS: Derivative log ratio spread; DNA: Deoxyribonucleic acid; FISH: Fluorescence *in situ* hybridization; POC: Product of conception; SNP: Single nucleotide polymorphism; STR: Short tandem repeat; UPD: Uniparental disomy; UPHD: Uniparental heterodisomy; UPID: Uniparental isodisomy.

## Competing interests

SB, BS, FS, JP, MS and CNSH declare no conflict of interests. AC and MB are employees of BlueGnome Ltd., an Illumina company.

## Authors’ contributions

SB outlined the study, participated in array design and analysis and drafted the manuscript. BS, FS, JP and MS carried out hybridizations, acquisition of data, interpretation and documentation. AC and MB designed the array platform and developed the analysis algorithm. CNSH supervised the study and finally approved the manuscript to be published. All authors read and approved the final manuscript.
